# Phase I-II study of ADXS31-142 alone and in combination with pembrolizumab in patients with previously treated metastatic castration-resistant prostate cancer (mCRPC): the KEYNOTE-046 trial

**DOI:** 10.1186/2051-1426-3-S2-P153

**Published:** 2015-11-04

**Authors:** Naomi B Haas, Mark N Stein, Ronald Tutrone, Rodolfo Perini, Andrew Denker, David Mauro, Lawrence Fong

**Affiliations:** 1 Abramson Cancer Center, University of Pennsylvania, Philadelphia, PA, USA; 2 Rutgers Cancer Institute of New Jersey, New Brunswick, NJ, USA; 3 Chesapeake Urology Research Associates, Baltimore, MD, USA; 4 Merck, Sharp & Dohme, Corp, North Wales, PA, USA; 5 Advaxis, Inc., Princeton, NJ, USA; 6 Department of Medicine, University of California San Francisco Helen Diller Family Comprehensive Cancer Center, San Francisco, CA, USA

## Background

Activation of tumor antigen-specific T-cell responses and bypass/neutralization of tumor immune tolerance mechanisms are essential for immunotherapeutic efficacy. *Listeria monocytogenes* (*Lm)*-listeriolysin O (LLO) immunotherapies have shown to both generate antigen-specific T-cell responses and neutralize cells that protect the tumor microenvironment. ADXS31-142 is a live attenuated *Lm*-LLO immunotherapy designed to target the prostate-specific antigen (PSA) and bioengineered to secrete an antigen-adjuvant fusion protein, consisting of a truncated fragment of the LLO fused to human PSA. Programmed death receptor 1 (PD-1) is a cell surface protein receptor central to T-cell immunity inhibition. Pembrolizumab is a humanized monoclonal antibody that binds to the PD-1 receptor, blocking interaction with PD-1 ligands and thereby inhibiting tumor immune evasion mechanisms. The combination of an *Lm*-LLO immunotherapy with an anti-PD-1 antibody has shown synergistic antitumor activity in preclinical studies.

## Methods

This is a Phase I-II, open-label, multicenter, nonrandomized, 2-part study (NCT02325557) in patients with mCRPC. Part A is a dose escalation of monotherapy ADXS31-142, followed by Part B, a dose escalation of ADXS31-142 plus pembrolizumab; Part B is followed by a dose-expansion cohort. Primary objectives are to evaluate the safety and tolerability of ADXS31-142 alone and in combination with pembrolizumab, and establish the ADXS31-142 recommended Phase II dose (RP2D) for monotherapy and combination therapy. Secondary objectives include evaluation of antitumor activity and progression-free survival. Male patients (≥18 years) with progressive mCRPC (≤3 prior systemic treatment regimens), on androgen deprivation therapy, and with an Eastern Cooperative Oncology Group (ECOG) performance status of 0–1 are eligible. Each dose-determining part will enroll up to 21 patients. ADXS31-142 and pembrolizumab (200 mg) are administered once every 3 weeks in a 12-week treatment cycle, with ADXS31-142 administration limited to weeks 1, 4, and 7. ADXS31-142 dose-escalations (up to a maximum of 1×10^10^ colony-forming units [CFU]) and de-escalations are done according to predefined dose-limiting toxicity criteria by applying the modified toxicity probability interval design. In Part A, the starting ADXS31-142 dose is 1×10^9^ CFU, and in Part B is either 1 level below the RP2D established for monotherapy or 1×10^9^ CFU, if this was the maximum tolerated dose for monotherapy. Once the ADXS31-142 RP2D for combination with pembrolizumab is determined, the expansion cohort will open for enrollment. A total of 30 patients are planned for treatment at the RP2D. The study is currently open and patient accrual is in progress.

## Trial registration

ClinicalTrials.gov identifier NCT02325557.

